# Unlocking the hydrocarbon potential: Formation evaluation and petrophysical properties of the upper Triassic Kurra Chine Formation in Sarta Oil Field, Kurdistan Region, Northern Iraq

**DOI:** 10.1016/j.heliyon.2024.e25173

**Published:** 2024-01-24

**Authors:** Mahdi K. Aswad, Muhamed F. Omer, Srood F. Naqshabandi

**Affiliations:** Department of Earth Science and Petroleum, College of Science, Salahaddin University, Erbil, Kurdistan Region, Iraq

**Keywords:** Kurra chine formation, Sarta oil field, Formation evaluation, Petrophysical properties, Northern Iraq

## Abstract

The Upper Triassic Kurra Chine Formation in the Sarta oil field of the Kurdistan Region of Northern Iraq has garnered limited attention, notwithstanding the keen interest of numerous international oil companies in drilling wells within this geological epoch. This study endeavors to thoroughly investigate the Formation Evaluation and petrophysical properties of the Kurra Chine Formation in the production oil field, with a specific focus on Sarta Well-2 (S-2). The research incorporates diverse methods for formation evaluation and analysis of petrophysical properties, employing conventional wireline logs such as Gamm-Ray, Neutron, Density, Sonic, Resistivity, Caliper, and Bit size.

The research findings reveal that the thickness of the Kurra Chine Formation in S-2 is approximately 380 m. The pay zones of S-2 exhibit an average shale volume of 17 %. The dominant lithology in S-2 comprises Limestone, Dolomite, Anhydrite, Shale, and Sandstone. The average total porosity within the pay zones is determined to be 6 % in S-2. Furthermore, the average effective porosity in reservoir zones of the S-2 is estimated to be 5 %, while the average secondary porosity in these zones is found to be 6 % in S-2. The average permeability in the pay zones of the Sarta well is reported to be 30.6 millidarcy (mD). Additionally, the average water saturation in the pay zones is determined to be 35 % in S-2, whereas the average hydrocarbon saturation is estimated to be 45 % in S-2.

This study furnishes comprehensive descriptions and analyses of the formation evaluation and petrophysical properties of the Kurra Chine Formation in Northern Iraq, shedding light on the characteristics and potential of this oil-bearing formation.

## 1 Introduction

The Sarta Oil Field, situated in the Kurdistan region of Iraq, was discovered in 2012 by Sheveron Company and is currently producing approximately 5000 barrels per day. Spanning an area approximately 35 km northwest of Erbil City, the field has been under joint development with Genel Energy since 2020 [1].

Carbonate reservoir rocks play a pivotal role in oil production across OPEC countries and the Middle East. Assessing the petrophysical properties of these carbonate reservoirs is a focal point for oil companies aiming to ascertain the hydrocarbon volume within the reservoirs. Unlike clastic rocks, carbonate rocks' reservoir properties are characterized by heightened complexity, featuring heterogeneity in porosity and permeability attributed to clay distribution within reservoir units [2]. The presence of dispersed clay types significantly influences the connectivity of carbonate reservoirs [3]. Moreover, heterogeneity in carbonate rocks, including limestone and dolomite, can be ascribed to various factors such as lithology, mineralogy/chemistry, pore types and connectivity, and sedimentary facies [4].

In the oil fields of Northern Iraq, challenges arise in managing water and hydrocarbon saturations. Some oil production wells experience an increase in water volume and a decrease in hydrocarbon volume. Conducting formation evaluation and petrophysical properties analysis becomes crucial in identifying the main reasons for these fluid volume changes [5]. Well-logging, a widely used method, proves effective in identifying and characterizing the petrophysical properties of carbonate reservoirs and other subsurface formations like basalt traps [6]. The identification of reservoir quality and pay zones is achieved by integrating seismic data interpretation, well-logging data, and core data [7]. Additionally, carbonate pore types are identified through the integration of petrophysical tools and petrographic analysis [8]. Wireline logging tools play a crucial role in identifying hydrocarbon source rock and reservoir characterizations of the drilled wells [9,10].

The Kurra Chine Formation, a carbonate formation housing various carbonate lithologies, has demonstrated prolific hydrocarbon potential in drilled wells in Northern Iraq. Geologist Wetzel's initial description noted the presence of limestone, papery shale, and dolomitic limestone with a thickness of 834 m [11]. The lower contact of the Kurra Chine Formation is characterized by hematite with the GeliKhana Formation, while the upper contact is gradational with the Baluti Formation [12]. The Kurra Chine Formation has been drilled in several oilfields in Kurdistan, presenting subsurface lithology consisting of limestone, dolomite, evaporite, shale unit, and hematite lenses.

At the Sarta field, the Kurra Chine Formation is located at depths ranging from 4150 m to 4750 m, with a thickness of 375 m. The primary lithologies recognized within the formation are limestone, dolomite, anhydrite, shale, and sandstone.

While previous research studies on the Kurra Chine Formation primarily focused on describing its outcrop characteristics and depositional settings, there is a notable lack of detailed descriptions regarding the petrophysical properties of the Carbonate Rocks of Kurra Chine [13,14,15,16,12,17]. Although some researchers have provided descriptions of specific units within the Kurra Chine Formation, such as Unit A and microfacies, a comprehensive understanding of its petrophysical properties remains elusive [18,12]. The Kurra Chine Formation displays various fractures and porosity types, including microtextures, joints, veins, and shelter fractures, with the GR method utilized for identifying lithofacies enhancement in clastic sedimentary rocks [5,19].

Despite these contributions, there is a considerable gap in detailed descriptions of the petrophysical properties of the Carbonate Rocks of Kurra Chine. Limited research offers a comprehensive understanding of the petrophysical properties and reservoir characterization of this formation in Northern Iraq. The current study aims to address this gap by providing a detailed analysis of the complete range of petrophysical properties. Additionally, it conducts a formation evaluation of the Kurra Chine Formation in a representative oil well within the Iraqi Kurdistan Region. The primary research question of this study is to comprehensively evaluate the petrophysical properties of the Kurra Chine Formation within a production oil field in Northern Iraq. The results of this research will contribute to filling a long-standing knowledge gap in Northern Iraqi oil wells, offering insights into water volume within production wells and the permeability characteristics of the reservoir zones. This project holds significant importance as it aims to comprehensively analyze all aspects of the Kurra Chine Formation, enabling a better understanding of effective porosity within the reservoir and the influences of shale on reservoir units.

## 2 Geological and tectonic setting of the study area

The Kurdistan region is situated within the Zagros fold belt, which extends along the Turkish-Iranian border and is renowned for its abundant hydrocarbon resources [20]. The Kurra Chine Formation, initially documented by Ref. [21] at the Ora fold outcrops near the Amedy district in Northern Iraq, is a notable geological feature in this region [21]. reported that the thickness of the Kurra Chine Formation at its type locality is 834 m. This formation is predominantly composed of limestone exhibiting dark brown and black colors, featuring thin to thick bedding of dolomite and breccia beds. The study area is located within the high folded zone, forming part of the broader Zagros fold belt. The subsurface analysis of the Sarta well is associated with the Pirmam Anticline in Erbil, along with the structural investigation of the subsurface.

The Sarta field, a recently discovered oil field, commenced production at the end of 2020 with an initial output of 8000 barrels per day (b/d). However, production has been adversely affected by water conning issues, leading to a decline to 3000 b/d. Additionally, completion problems with the boreholes have contributed to reduced production.

The tectonics of the studied area involve the interaction between the Arabian and Eurasian plates. Iraq is situated in the Northeastern region of the Arabian Plate, which collided with the Iranian Plate, giving rise to the formation of a foreland basin known as the Zagros fold belt. According to Ref. [22], Iraq was part of the Gondwana Continent during the Paleozoic Era, alongside the Indian and African plates. Rifting and extension occurred during the middle Permian to the Triassic and Jurassic periods, leading to the separation of the Gondwana continent and the creation of the NeoTethys Ocean [23]. Carbonate rocks were the primary sedimentary rocks formed during this period, while clastic rocks originated from fluvial deltaic depositional environments [24]. The thickness of Neotethys reached its peak at 4000 km during the Late Triassic and Middle Jurassic periods [25].

**Fig. 1:**
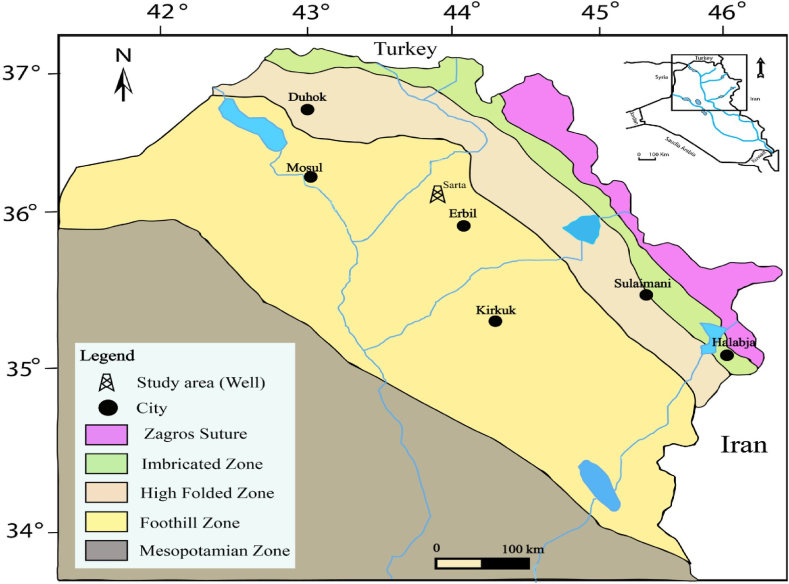
The location map of the studied area [27]. It displays the Iraqi region and highlights the location of the studied borehole in the Foothill Zone.

The ongoing research aims to investigate the petrophysical properties of a specific subsurface section within the Sarta Oil Field (Fig. 1), located at coordinates 430 58′ 20.399″ E 360 31′ 39.228″ N. The Kurra Chine Formation underlies the Baluti Formation and overlies the Galikhana Formation. The lower contact of the Kurra Chine Formation is unconformable, while its upper contact is conformable [26].

## 3 Material and methodology

The research undertaken involved the comprehensive analysis of conventional wireline logs obtained from a single well within an oil field located in the Kurdistan region of Iraq. Specifically, the focus was on Subsurface logging data related to the Upper Triassic Kurra Chine Formation. The wireline log dataset included crucial measurements such as Gamma Ray (GR), Caliper (Cal), Bit size (BS), Neutron Porosity (ND), Bulk Density (BD), Acoustic log (AL), Resistivity logs (RL), and Photoelectric tool (PhT). These advanced tools were deployed with the primary objective of discerning essential petrophysical properties associated with the Kurra Chine Formation. These properties include lithology, total and effective porosity, secondary porosity, shale volume, permeability, water saturation, and hydrocarbon saturation.

The extracted data from the Las file was meticulously inputted into the esteemed Schlumberger Tech-Log software. This step facilitated a thorough analysis of reservoir rock properties that are intrinsic to the Kurra Chine Formation. The utilization of sophisticated tools and advanced software enhances the accuracy and depth of the study, providing valuable insights into the geological characteristics and hydrocarbon potential of the formation.

### 3.1 Shale volume from GR

In porous reservoirs, shale volume plays a significant role as it represents one of the heterogeneous materials that occupy the reservoir pore spaces. To assess shale volume within the reservoir, various techniques are employed through the utilization of Tech-Log software. These techniques include shale volume determination using methods such as (Neutron-Density, Neutron-Sonic, Density-Sonic, and GR). According to Ref. [28], Linear GR is considered a rapid and straightforward approach for quantifying shale volume within a porous reservoir Table 1. This is a shale volume formulas

1 IGr = {GRlog-GRmin}/{GRmax-GRmin}

2 Vsh = 0.33*(2^2^*^IGR^ -1)

**Table 1:** Parameters used for identifying shale from Gamma Ray and N-D methods.

Well	Zone	Top	Bottom	GR_Matrix	GR_Shale	NPHI Shale	NPHI fluid	RHOB_Matrix	RHOB_shale	RHOb_fluid
Sarta-2	All	3460 m	3839 m	10 API	100 API	0.4 %	1 %	2.65 g/cm^3^	2.45 g/cm^3^	1 g/cm^3^

Vsh = Shale volume, GRmin = minimum GR (reservoir zone), GRmax = maximum GR (shale zone), GRlog = GR from log.

### 3.2 Lithology

Two major methods for lithological identification are M and N cross plots and N-D cross plots.

3 M = {tf-tlog}/(ρb-pf}

4 N = {∅Nf- ∅N}/{ ρb-pf}

tf = transit time of fluid (fresh mud = 189), tlog = transit time from the log-in tech-log

ρb = bulk density from log-in tech-log, pf = density of the fluid (freshwater = 1 and for saline water = 1.1).

∅Nf = neutron porosity of the fluid = 1, ∅N = neutron porosity form in tech-log.

### 3.3 Porosity

Porosity refers to the proportion of bulk volume to the pore volume [2]. Wireline logs can identify the total, effective, and secondary porosity.

#### 3.3.1 Total porosity

The total porosity for the current study was estimated from different porosity logs and cross plots including neutron, density, sonic, and N-D cross plots [25].

Total porosity from Density log

5 ∅D = {ρma-ρb}/{ρma-ρf)

ρma = matrix density (depending on lithology), ρb = bulk density from the log

ρf = fluid density (1 for fresh and 1.1 for saline water).

Total porosity from Sonic log

6 ∅S = {tlog – tma}/{tf-tma}

tf = transit time of fluid (fresh mud = 189).

tlog = transit time from the log-in tech-log

tma = matrix transit time (depending on lithology).

Total porosity from Neutron – Density (N-D) logs.

Neutron logs work by emitting high-energy neutrons into the formation, and these neutrons interact with the atomic nuclei in the rocks. The number of neutrons that return to the detector depends on the density of the formation and the amount of hydrogen present. Porosity is indirectly inferred from the neutron log by assuming that the hydrogen content in the formation is primarily related to the presence of fluids (usually water) in the pore spaces. By measuring the neutron response and making certain assumptions, such as the matrix and fluid properties, it is possible to estimate porosity.

Total porosity from N-D cross plots

7 ∅T = {∅N+∅D}/2

#### 3.3.2 Effective porosity

Effective porosity is the main active part of the reservoir which will be calculated for the accumulation of hydrocarbon. Effective porosity can be estimated by the below formula.

8 ∅E = ∅T * (1-Vsh)

#### 3.3.3 Secondary porosity or fracture porosity

Secondary porosity is the main porosity in carbonate rocks. Carbonate rocks contain high fractures due to the dissolution of carbonate materials. It can be estimated by the below formula.

9 ∅f = ∅ _N-D_ - ∅S

∅f = fracture porosity or secondary porosity, ∅ _N-D_ = Neutron and density porosity.

∅_S=_Sonic Porosity.

### 3.4 Permeability

Permeability can be estimated in the following equation in wireline analysis.

Permeability from Wyllie-Rose

10 K = {100 ∅ ^2.25^/ Swi}^2^

Permeability from Timur

11 K = 0.136 *{∅^4.44^/Swi}^2^

Where,

K = permeability, ∅ = formation porosity, Swi = irreducible water saturation.

### 3.5 Water saturation

The estimation of water saturation relies on the lithology of the formation [5]. The Kurra Chine Formation consists of both clean formations and shale formations. Two methods are utilized to estimate water saturation, one for clean lithologies and the other for shale zones.

#### 3.5.1 Archie equation 1942

This method is employed to asses water saturation in reservoirs where shale content is less than 15 %. It is used for carbonate and clastic reservoirs based on shale content. This equation is suitable for the Kurra Chine Formation due to the existence of many active reservoir zones [29].

12 Sw = {(F*Rw)/Rt}-n

13 F = a/∅m

#### 3.5.2 Simandoux equation 1963

This equation is used for shale zones. If any zones contain shale of more than 10 % then it can work. Kurra Chine Formation has some units of shale, this method is applicable to estimate water saturations from shale units and stratus.

14 Sw = {(-Vsh/Rsh) +/− √(Vsh/Rsh)^2^ + (4∅m/wart)}/(2∅^2^/aRw)

Sw = water saturation.

F= Formation factor, Rw = Formation water resistivity from the cross-plot.

Rt = True formation resistivity from the log, ∅ = Formation Porosity

m and n = cementation factor for carbonate rocks is 2, a = 1 it is a constant.

#### 3.5.3 Waxman Smith equation 1968

15 SW = {(-Vsh/Rsh) +/−√ (Vsh/Rsh) 2 + (4ɸm/wart)}/ (2ɸ 2 /aRw)

Where,

Sw = water saturation; Vsh = Shale volume; Rsh = Resistivity of shale; M cementation factor = 2; Rw = formation water resistivity; RT = true formation resistivity; ɸ = formation porosity; a = is constant = 1.

### 3.6 Hydrocarbon saturation

Hydrocarbon saturation is crucial for oil companies to evaluate hydrocarbon in the reservoir [5]. After identifying water saturation, hydrocarbon saturation is extracted from unity.

16 Sh = 1-Sw

Hydrocarbon saturation is divided into two parts, namely movable and residual hydrocarbon saturation.

#### 3.6.1 Movable hydrocarbon saturation

Movable hydrocarbon saturation includes the volume of oil that can be estimated in the reservoir.

17 MSh = {Sxo- Sw}* ∅E

#### 3.6.2 Residual hydrocarbon saturation

Residual hydrocarbon saturation includes the oil ratio that will remain in the reservoir.

18 RSh = (1- Sxo)

Sh = Hydrocarbon Saturation, Sw = Water saturation, MSh = Movable Hydrocarbon Saturation, Sxo = Flushed zone water saturation, ∅E = Effective porosity, RSh = Residual Hydrocarbon Saturation.

## 4 Results and discussion

Wireline logging in the oil and gas industry uses various equations and mathematical models to achieve several objectives, including:

Formation Evaluation: The primary objective of wireline logging equations is to evaluate the properties of subsurface formations. These properties can include porosity, permeability, lithology (rock type), fluid saturations (oil, water, gas), and more. Equations are used to convert raw logging data into meaningful information about the subsurface geology and hydrocarbon reservoirs.

Reservoir Characterization: Wireline logging equations help characterize the reservoir's physical and petrophysical properties. This information is essential for reservoir engineers and geoscientists to understand the reservoir's potential, its capacity to store hydrocarbons, and its ability to produce them economically.

Hydrocarbon Identification: Equations can help identify the presence of hydrocarbons (oil and gas) and distinguish them from other fluids like water and brine. This is crucial for assessing the commercial viability of a reservoir.

Wellbore Stability: Logging equations are used to assess the stability of the wellbore, including evaluating the mechanical properties of the surrounding rock formations. This information is vital for drilling operations to ensure well integrity and safety.

Fluid Flow Modeling: Equations can be used to model fluid flow within the reservoir, which is essential for predicting production behavior, optimizing well placement, and designing enhanced recovery techniques.

Reservoir Monitoring: Wireline logging equations are also used for monitoring reservoir changes over time. By repeatedly logging the same well, engineers can track reservoir depletion, fluid movement, and the effects of production operations.

Quality Control: Equations are used for quality control and data validation. They help identify and correct any errors or anomalies in the logging data to ensure accurate interpretation.

Decision-Making: Ultimately, the data obtained through wireline logging and the associated equations inform critical decisions about drilling, completion, and production strategies. They help operators make informed choices to maximize the economic recovery of hydrocarbons.

### 4.1 Shale volume from GR and larionov

**Fig. 2:**
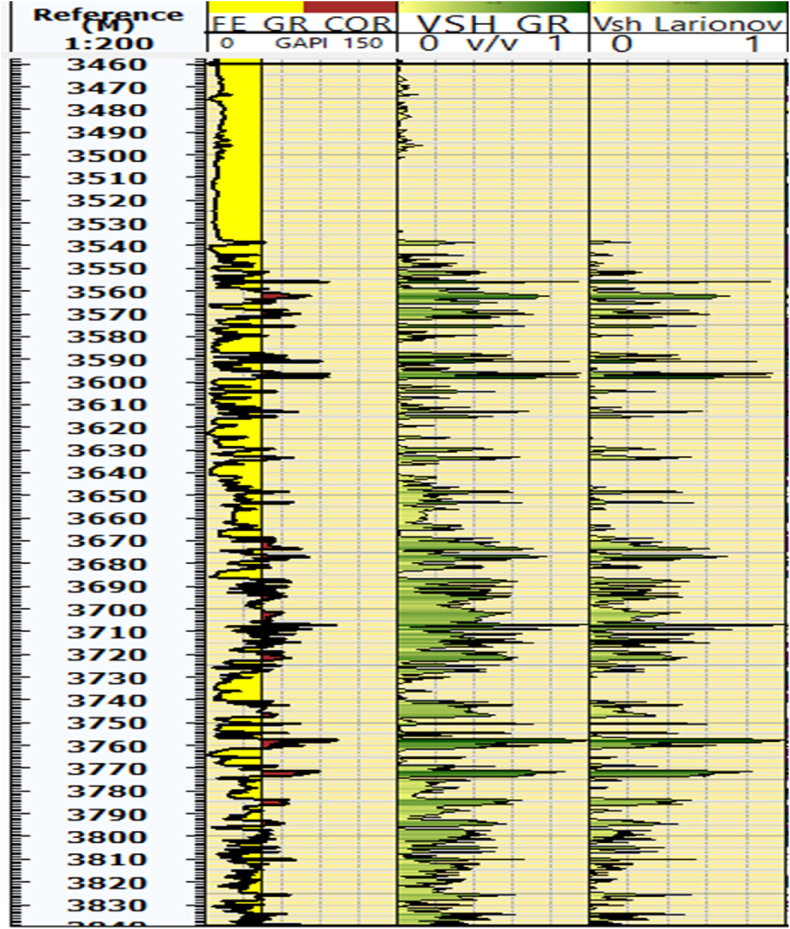
The borehole depth, GR, and shale volume information from both the GR method and the shale volume from the Larionov method. The figure specifically highlights.

Estimating shale volume is a crucial step in petrophysical interpretation and Formation Evaluation due to its significant impact on reservoir units, particularly porosity, and permeability. The Gamma Ray method is considered one of the best and fastest approaches for shale volume estimation. The shale volume has been corrected using the [30] equation. In the Kurra Chine Formation of the Sarta Oil field, shale percentages vary. Clean zones exhibit low shale content, while other zones contain shale of approximately 60 % such as a depth of 3560–3565 and 3670–3770 m (Fig. 2).

the presence of shale at different depths using both GR and Larionov methods.

### 4.2 Lithology from neutron density and M and N cross-plots

**Fig. 3a:**
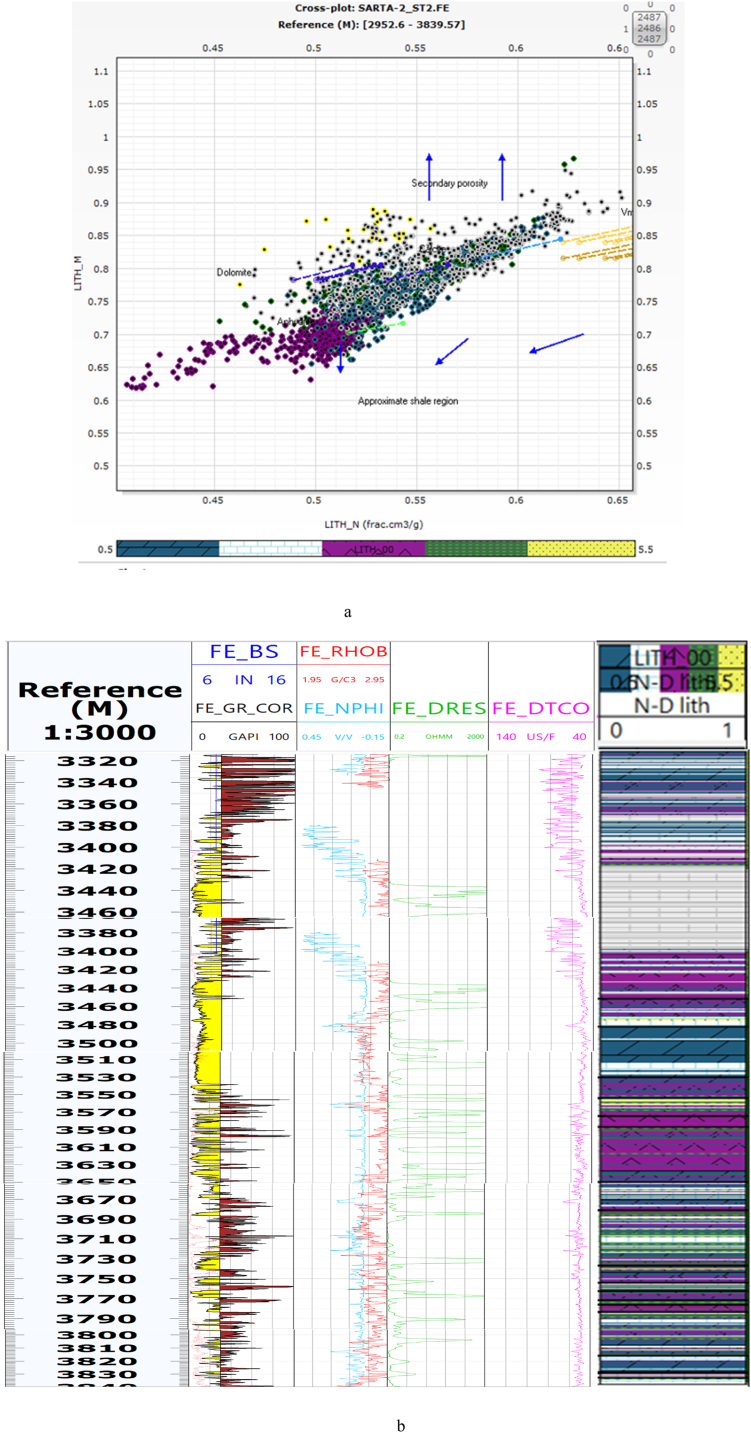
The cross plots between parameters labeled as (M and N) that are used for identifying the lithology of the Sarta oil field. The caption further clarifies that the bottom legend provides information about the lithology types represented in the figure, starting from the left side, namely (dolomite, limestone, anhydrite, shale, and sandstone). These lithology types are the main ones found in the wireline logs. Fig. 3b: The cross plots between parameters labeled as (Neutron and Density) that are utilized for identifying the lithology of the Sarta oil field. Additionally, the caption explains that this figure represents a composite log consisting of GR, neutron, density, resistivity, and sonic log. The column on the right side of the figure provides information about the lithology types based on cross-plots of (N–D). The figure illustrates the thickness of each lithology type, namely dolomite, limestone, anhydrite, shale, and sandstone, which are the primary lithologies found in the wireline logs.

Lithology identification is a main part of the Formation Evaluation of Carbonate Rocks. N-D and M and N were used to identify the main lithology in both Oil fields. The main lithologies in the Sarta Oil field are Anhydrite, Dolomite, Limestone, Shale, and Sandstone (Fig. 3a and b). The clustering method through wireline logging is a new technique to identify rock types [31].

### 4.3 Porosity

#### 4.3.1 Total porosity from density, sonic, and (N -D) cross plots

**Fig. 4:**
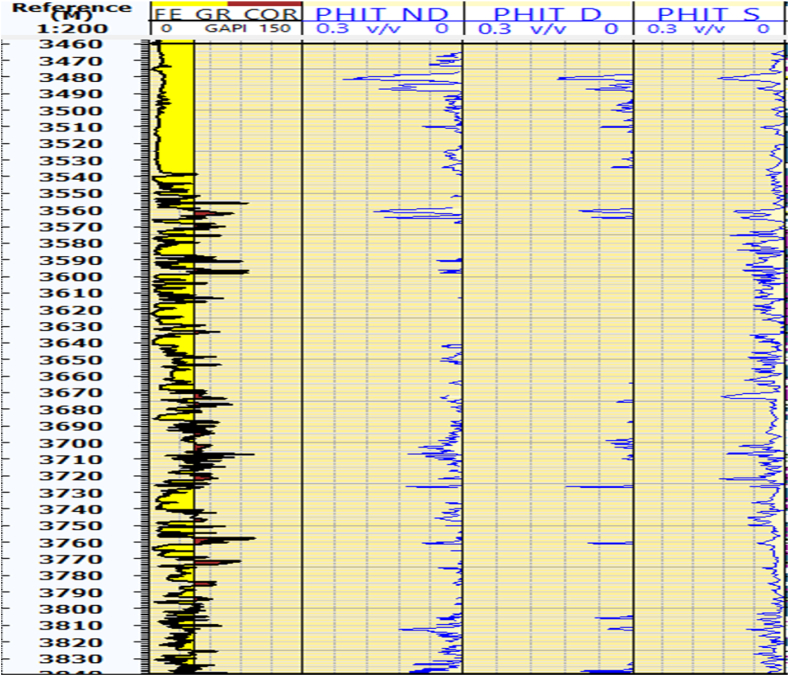
The cross plots of neutron-density (PHIT_ND), density log (PHIT_D), and sonic log (PHIT_S), specifically focusing on the measurement of total porosity. The caption further explains that the figure shows the total porosity at each depth for the Sarta oil field using these three logs.

Porosity is a primary petrophysical parameter that significantly affects hydrocarbon accumulations within the reservoir. The reservoir units within the Kurra Chine Formation exhibits high porosity. In the Sarta Oil field, the N-D logs indicate a porosity of 18 %, whereas the Density and Sonic logs show a porosity of 12 % in the reservoir zones (Fig. 4). The average porosity in reservoir units of the Sarta Oil field is 6 %.

#### 4.3.2 Effective porosity from density, sonic, and neutron density cross plot

**Fig. 5:**
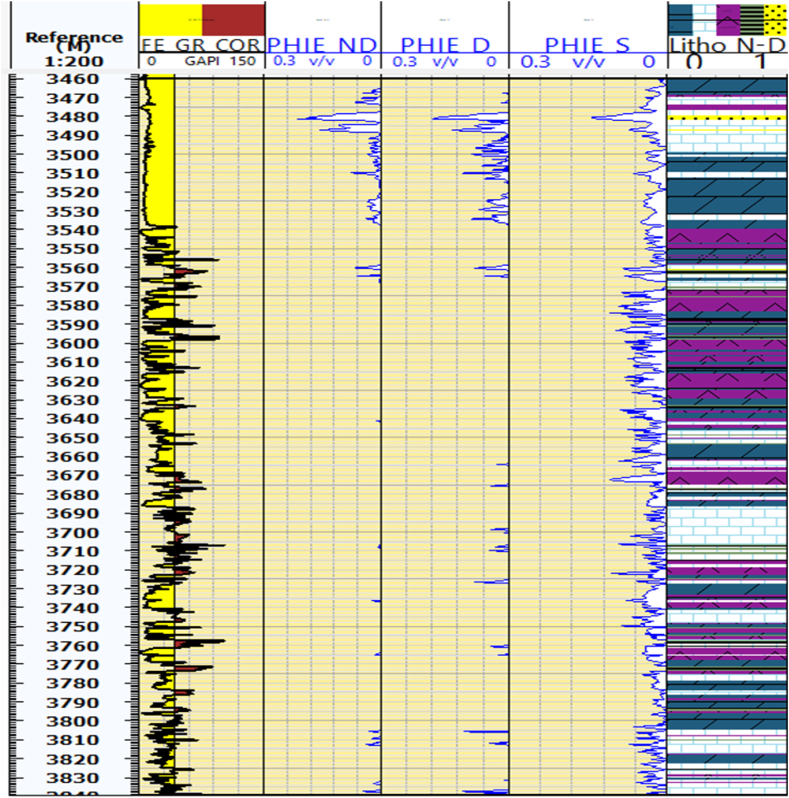
The cross plots of neutron-density (PHIE_ND), density log (PHIE_D), and sonic log (PHIE_S), specifically focusing on the measurement of effective porosity. The caption further explains that the figure shows the effective porosity at each depth for the Sarta oil field using these three logs.

The Kurra Chine Formation is a significant Carbonate rock formation of the Triassic period. Effective porosity plays a crucial role in the distribution and migration of hydrocarbon within the reservoir. Limestone and Sandstone units exhibit high effective porosity across all tools when compared to dolomite units (Fig. 5).

### 4.4 Fractural porosity and permeability

**Fig. 6:**
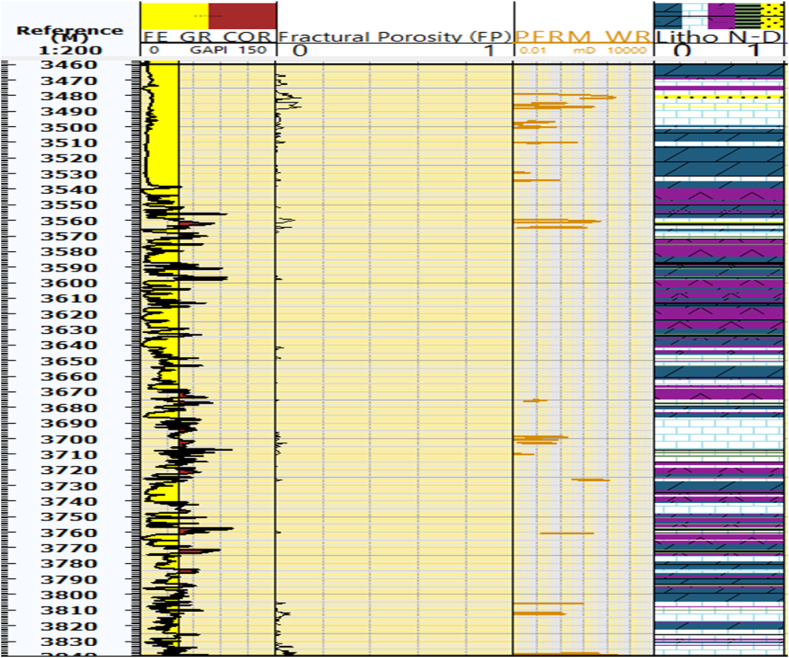
The fracture porosity or secondary porosity (FP), which is an important reservoir parameter in carbonate rocks. Additionally, the figure also displays the permeability, which is another crucial parameter for reservoir characteristics. The figure shows the distributions of secondary porosity and permeability across different lithology units at each depth.

The Kurra Chine Formation primarily comprises carbonate rocks, which are known to contain significant fractures. These fractures are a result of the diagenesis process that occurs within carbonate rocks. The sonic log is a key tool for identifying fractural porosity or secondary porosity. Fractural porosities are predominantly observed in Limestone and Dolomite within the Sarta Oil Field. The Kurra Chine Formation in the Sarta oil field recorded an 8 % in Sarta (Fig. 6).

Permeability is the second primary parameter in the petrophysical properties of Carbonate Reservoirs. It controls the flow of hydrocarbon through the reservoir. Standard well logs provide a foundation for estimating permeability, incorporating additional measurements like core data, pressure transient testing, dielectric measurements, NMR logs, and image logs that can help refine and improve permeability quantification in subsurface reservoirs. Combining various data sources allows for a more comprehensive understanding of reservoir properties. The Kurra Chine Formation exhibits significant permeability in its Limestone units, with values reaching approximately 100mD in the Sarta Oil Field (Fig. 6).

### 4.5 Water saturation and hydrocarbon saturation

Water saturation in pores undergoes dynamic changes during and after deposition. Initially, during the deposition process, water saturation in pores is fully saturated due to the aqueous environment. Following deposition, these pores are influenced by the diagenesis process, but water saturation typically remains at 100 %. However, when hydrocarbon enters the pores, water saturation decreases as hydrocarbon displaces the water due to its lower density.

The estimation of water saturation in specific scenarios involves the use of mathematical models. For instance, in cases where water shale volume is less than 15 %, Archie's Equation is a suitable method for determining water saturation. Additionally, for shale volumes less than 60 %, other equations referenced in the literature [24,32] are employed. Water saturation enhances the compressional wave velocity and reduces shear velocity inside the porous media [10]. These equations and models play a crucial role in accurately assessing and understanding water saturation in reservoir rocks, particularly in the context of hydrocarbon exploration and production.

**Fig. 7:**
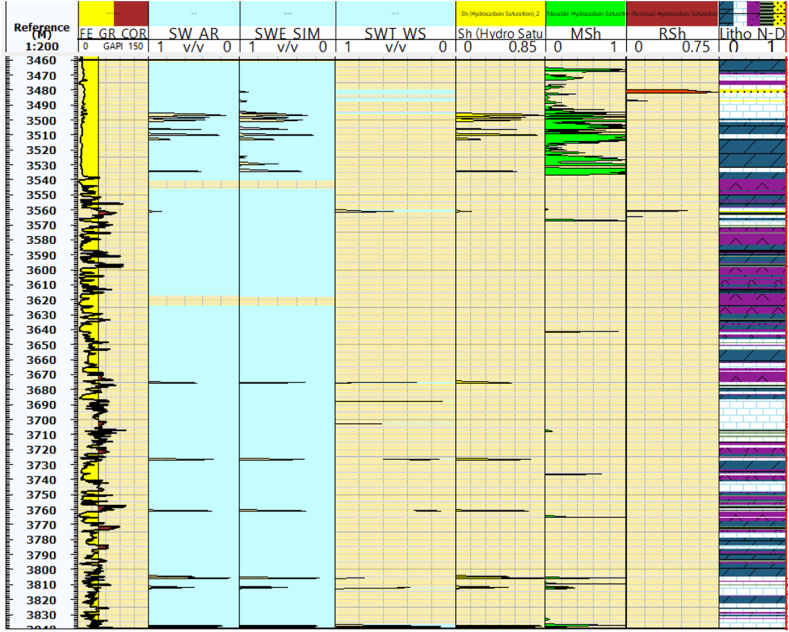
The water saturation (Sw) values calculated using Archie's, Simandoux, and Waxman Smith equations. Additionally, it presents hydrocarbon saturations (Sh), especially movable and residual hydrocarbon saturation. The caption clarifies that the yellow column represents the hydrocarbon saturation, while the green column indicates the movable hydrocarbon saturation. Furthermore, the figure illustrates the water and hydrocarbon saturation values for each depth, along with the known lithologies associated with those depths. (For interpretation of the references to color in this figure legend, the reader is referred to the Web version of this article.)

In the specific context of the Kurra Chine Formation in the Sarta Field, water saturation is estimated using different equations. According to Archie's, Simandoux's, and Waxman Smith's equations, the water saturation is approximately 70 %, 60 %, and 50 %, respectively (Fig. 7). These equations provide valuable insights into the distribution of water within the formation and are essential for reservoir characterization.

Hydrocarbon saturation, on the other hand, serves as a critical indicator of the presence of hydrocarbons in reservoir zones. In the Sarta Oil Field, Kurra Chine Hydrocarbon Saturation is visually represented by a yellow column, accounting for approximately 45 % of the data presented in (Fig. 7). This information is crucial for understanding the distribution and potential productivity of hydrocarbon reservoirs within the Kurra Chine Formation in the Sarta Field. The integration of water and hydrocarbon saturation data is vital for making informed decisions regarding reservoir management and production strategies.

## 5 Discussions

### 5.1 Shale volume

The upper part of the Kurra Chine Formation at depths ranging from 3460 to 3550 m contains extremely low shale volume. This area is considered the main reservoir zone in the Kurra Chine Formation. In contrast, the middle part at depths ranging from 3560 to 3565 m and the lower part at depths ranging from 3670 to 3770 m of the Kurra Chine Formation contain high shale volume. The average shale volume at the Sarta oil field is reported to be 20 %. The reservoir thickness, which refers to the thickness of the rock formation containing oil or gas is 22 %. The pay thickness, which represents the portion of the reservoir that is economically viable for production, is 17 % (Table 2).

**Table 2:** The Gross, Net, and Net to Gross values, along with the average results of all parameters. The table presents the final results of each log and tools analyzed using the Tech-Log software.

Flag name	Top M	Bottom m	Reference	Gross m	Net M	Not net m	Net to Gross	AV. Shale	Av. Porosity	Av. water Saturation	AV. Permeability mD
Rock	2952.5	3839.5	m	836.9	290.9	595.8	0.328	0.18	0.05	0.701	12.28
Res	2952.5	3839.5	m	836.9	111.5	267.4	0.126	0.20	0.05	0.701	12.28
Pay	2952.5	3839.5	m	836.9	2.5	367.2	0.003	0.17	0.06	0.359	30.62

### 5.2 Lithology

**Fig. 8:**
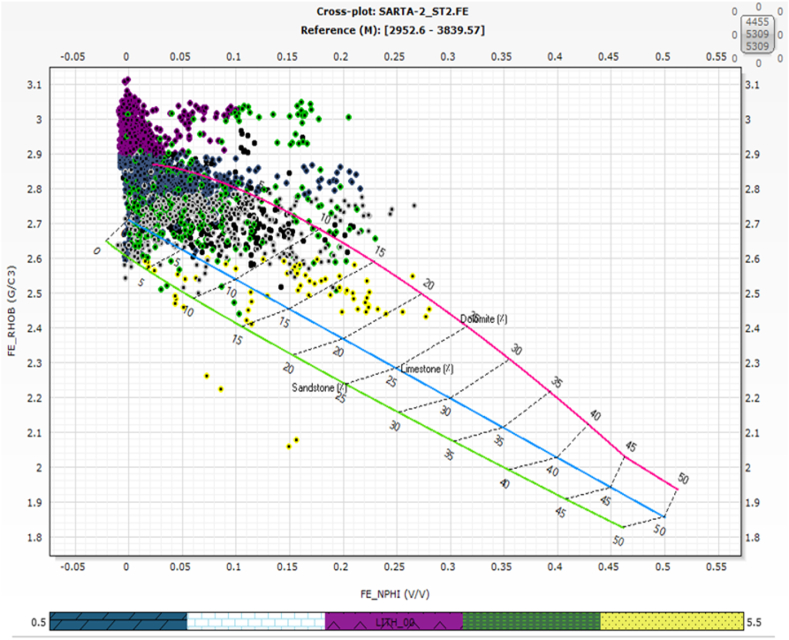
The cross plots between parameters labeled as (Neutron NPHI - Density RHOB) that are used for identifying the lithology of the Sarta oil field. The caption further clarifies that the bottom legend provides information about the lithology types represented in the figure, starting from the left side, namely (dolomite, limestone, anhydrite, shale, and sandstone). These lithology types are the main ones found in the wireline logs.

According to a previous study [18], the lithology of the Kurra Chine Formation was reported to primarily include Limestone, Shale, Dolomite, and Anhydrite. However, the findings of this present study reveal a slightly different composition for the Kurra Chine Formation in terms of lithology. Based on Wireline logs, the identified lithologies include Anhydrite, Limestone, Dolomite, Shale, and Sandstone. The distribution of lithology in the Sarta Oil field is visually represented in (Fig. 8), where N-D represents the lithology.

Porosity, a critical factor controlling reservoir properties, exhibits variation corresponding to lithology and shale content. In the Kurra Formation, porosity ranges from 2 % to 20 %. Specifically, in the Sarta Oil field, the upper part characterized by limestone lithology displays high porosity, reaching up to 18 %. This elevated porosity in the upper part of the Sarta Oil field is a notable feature.

Effective porosity, which is influenced by the pore throat characteristics of the reservoir, shows variations among different lithological units. Limestone and sandstone units consistently exhibit higher effective porosity across all measurement tools. The upper units of the Kurra Chine Formation, particularly those with limestone lithology, serve as the primary reservoir in the Sarta Oil field. These findings contribute valuable insights into the heterogeneity of lithology and its impact on porosity and effective porosity within the Kurra Chine Formation in the Sarta Oil field.

### 5.3 Fracture porosity and permeability

In the Sarta Oil Field, significant fractural porosities are notably observed in Limestone and Dolomite rocks. Furthermore, the average secondary porosity within the Kurra Chine Formation in the Sarta Oil field is determined to be 6 %. Permeability, a parameter crucial in determining the ease of fluid flow within the reservoir, exhibits a relatively low value in the Sarta Oil Field. The average permeability in the Kurra Chine Formation is reported to be 30.6mD, as indicated in the table.

The observed reduction in permeability is attributed to factors such as pore throat characteristics and flow connectivity within the reservoir. Notably, in the Sarta Field, there has been an increase in water volume after production, attributed to the widening of the fracture aperture in the pores. Consequently, the influx of water volume into the reservoir is expected to rise due to improved pore connectivity within the water zone. Understanding these permeability dynamics is crucial for effective reservoir management and production optimization in the Sarta Oil Field.

### 5.4 Water and hydrocarbon saturation

**Fig. 9:**
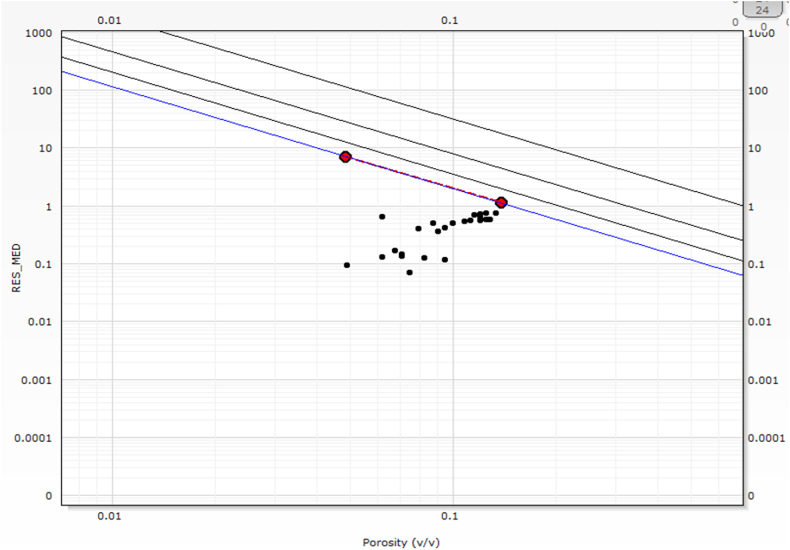
The Pickett plot which is a graphical method used to determine formation water resistivity (Rw) and cementation exponent (m) in carbonate rocks based on porosity and resistivity data.

The fluid dynamics within the reservoirs of the Sarta Oil Field are predominantly influenced by water and hydrocarbon saturations. Estimating water saturation in reservoir zones with shale volume below 15 % is achieved using Archie's equation, while Simandoux and Waxman Smith's equations are employed for shale zones. Within the Kurra Chine Formation, notably high water saturation is observed in the Limestone and Dolomite units. The average water saturation in the Sarta Oil Field is reported to be 35 %, as shown in (Table 2). The field has encountered challenges with increasing water saturation post-production for several months. Wireline log data indicates a significant volume of water present in the field, with the potential for further increases due to the opening of fractures. Conversely, the Kurra Chine Formation exhibits high water saturation in both the upper and lower parts of the formation, albeit at a low percentage. Picket plots were employed for water resistivity (Rw) and cementation factor m, as illustrated in (Fig. 9).

**Fig. 10:**
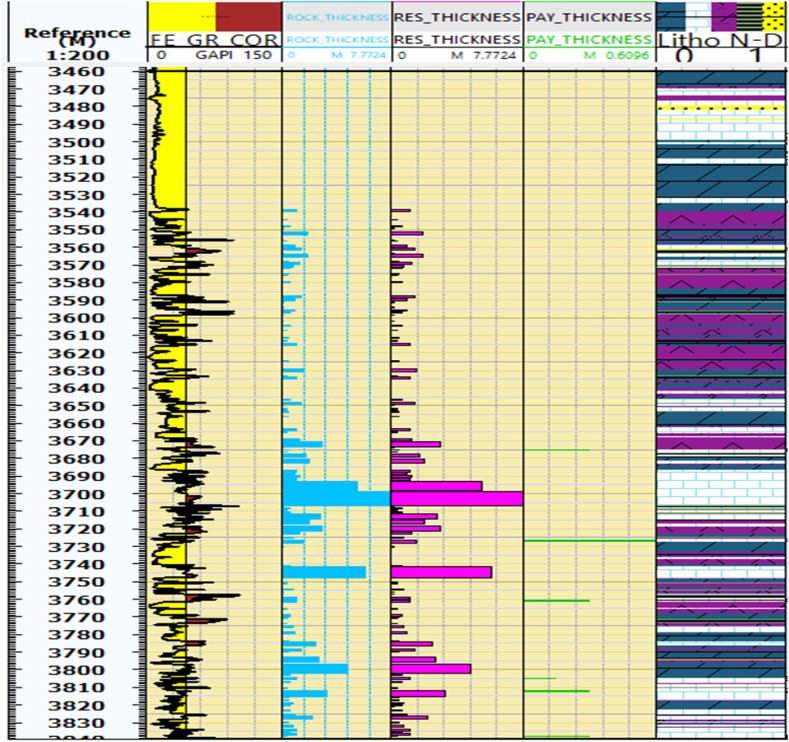
The distributions of rock thickness, reservoir thickness, and pay thickness. It further explains that the figure specifically focuses on the main reservoir thickness and pay thickness in limestone lithology. The figure provides information on the lithology associated with depth.

In terms of hydrocarbon saturation, the average value in the reservoir zones of the Kurra Chine Formation within the Sarta Field is determined to be 45 %, as depicted in (Fig. 7). The rock thickness, reservoir thickness, and pay thicknesses vary from lithology to lithology. The pay zones primarily consist of limestone rocks, as depicted in (Fig. 10). The main cut-off parameters are elaborated in Table 3, providing valuable insights into the hydrocarbon potential and distribution within the Kurra Chine Formation in the Sarta Oil Field.

**Table 3:** The cut-off parameters used in Tech-Log.

Well	Zone	Top	Bottom	gross	Shale Min	Shale Max	Porosity Min	Porosity Max	Water Saturation Min	Water Saturation Max	Permeability Min	Permeability Max
Sarta-2	All	3460	3839	836.9	0 v/v	0.7 v/v	0 v/v	0.5 v/v	0 v/v	0.85 v/v	0 Md	10000 md

## 6 Conclusions

Based on the analysis of wireline logs from the Sarta-2 well in Northern Iraq, the following conclusions can be drawn.

• The Kurra Chine Formation exhibits 380 m thick in the Sarta oil field (S-2).
• The average shale volume in the pay zones of the Sarta oil field is 17 %.
• The main lithology in Sarta Oil Field consists of carbonate rocks such as Limestone and Dolomite, as well as Evaporite rock (Anhydrite) and Clastic rocks (Shal and Sandstone).
• The average total porosity in pay zones of Sarta Oil Field is 6 %, including heterogeneous reservoirs with varying pore space.
• The average effective porosity in reservoir zones of the Sarta Oil Field is 5 %. This lower effective porosity is attributed to carbonate diagenesis and pore filling through mineralization in the late stage of carbonate rock formation.
• The average secondary porosity in Sarta Oil Field reservoir zones is 6 %, particularly evident in dolomite and limestone reservoir units.
• The average permeability in pay zones of Sarta Oil Field is 30.6mD, indicating low permeability due to the presence of disconnected pore throats in carbonate rocks.
• The average water saturation in the pay zones of the Sarta Oil Field is 35 %, suggesting that the Sarta field has a higher water content.
• The average hydrocarbon saturation in the pay zones of the Sarta Oil Field is 45 %, indicating water occupies a significant portion of the pores in the Sarta pores.

In summary, the study provides valuable insights into the formation evaluation and petrophysical properties of the Kurra Chine Formation in Northern Iraq, highlighting variation in lithology, total and effective porosity, permeability, water, and hydrocarbon saturations content in the Sarta oil field. These findings contribute to a better understanding of the reservoir characteristics and potential in these oil-bearing formations.

## CRediT authorship contribution statement

**Mahdi K. Aswad:** Writing – original draft, Software. **Muhamed F. Omer:** Investigation, Formal analysis. **Srood F. Naqshabandi:** Writing – review & editing, Project administration.

## Declaration of competing interest

The authors declare that they have no known competing financial interests or personal relationships that could have appeared to influence the work reported in this paper.
